# PTTG expression in different experimental and human prolactinomas in relation to dopaminergic control of lactotropes

**DOI:** 10.1186/1476-4598-6-4

**Published:** 2007-01-12

**Authors:** Carolina Cristina, Graciela S Díaz-Torga, Rodolfo G Goya, Sham S Kakar, María I Perez-Millán, Vanessa Q Passos, Daniel Giannella-Neto, Marcello D Bronstein, Damasia Becu-Villalobos

**Affiliations:** 1 Instituto de Biología y Medicina Experimental, Consejo Nacional de Investigaciones Científicas y Técnicas. V. Obligado 2490. (1428) Buenos Aires. Argentina; 2 Institute for Biochemical Research-Histology B, Faculty of Medicine, University of La Plata, Argentina; 3 Department of Medicine, James Graham Brown Cancer Center. University of Louisville, Louisville, KY, USA; 4 Neuroendocrine Unit, Division of Endocrinology and Metabolism, Hospital das Clinicas. University of Sao Paulo Medical School, Sao Paulo, Brazil; 5 Laboratory for Cellular and Molecular Endocrinology (LIM 25), Hospital das Clinicas. University of Sao Paulo Medical School, Sao Paulo, Brazil

## Abstract

**Background:**

Pituitary tumor transforming gene (pttg) is a novel oncogene that is expressed at higher level in most of the tumors analyzed to date compared to normal tissues. Nevertheless, its expression in prolactinomas and its relation with the pituitary dopamine receptor 2 (D2R) are not well defined. We sought to determine the pituitary level of pttg in three different experimental models of prolactinomas with altered dopaminergic control of the pituitary: the dopaminergic D2R knockout female mouse, the estrogen-treated rat, and the senescent female rat. These three models shared the characteristics of increased pituitary weight, hyperprolactinemia, lactotrope hyperplasia and reduced or absent dopaminergic action at the pituitary level. We also studied samples from human macroprolactinomas, which were characterized as responsive or resistant to dopamine agonist therapy.

**Results:**

When compared to female wild-type mice, pituitaries from female D2R knockout mice had decreased PTTG concentration, while no difference in pttg mRNA level was found. In senescent rats no difference in pituitary PTTG protein expression was found when compared to young rats. But, in young female rats treated with a synthetic estrogen (Diethylstylbestrol, 20 mg) PTTG protein expression was enhanced (*P *= 0.029). Therefore, in the three experimental models of prolactinomas, pituitary size was increased and there was hyperprolactinemia, but PTTG levels followed different patterns.

Patients with macroprolactinomas were divided in those in which dopaminergic therapy normalized or failed to normalize prolactin levels (responsive and resistant, respectively). When pituitary pttg mRNA level was analyzed in these macroprolactinomas, no differences were found.

We next analyzed estrogen action at the pituitary by measuring pituitary estrogen receptor α levels. The D2R knockout female mice have low estrogen levels and in accordance, pituitary estrogen receptors were increased (*P *= 0.047). On the other hand, in senescent rats estrogen levels were slightly though not significantly higher, and estrogen receptors were similar between groups. The estrogen-treated rats had high pharmacological levels of the synthetic estrogen, and estrogen receptors were markedly lower than in controls (*P *< 0.0001). Finally, in patients with dopamine resistant or responsive prolactinomas no significant differences in estrogen receptor α levels were found. Therefore, pituitary PTTG was increased only if estrogen action was increased, which correlated with a decrease in pituitary estrogen receptor level.

**Conclusion:**

We conclude that PTTG does not correlate with prolactin levels or tumor size in animal models of prolactinoma, and its pituitary content is not related to a decrease in dopaminergic control of the lactotrope, but may be influenced by estrogen action at the pituitary level. Therefore it is increased only in prolactinomas generated by estrogen treatment, and not in prolactinomas arising from deficient dopamine control, or in dopamine resistant compared with dopamine responsive human prolactinomas. These results are important in the search for reliable prognostic indicators for patients with pituitary adenomas which will make tumor-specific therapy possible, and help to elucidate the poorly understood phenomenon of pituitary tumorigenesis.

## Background

Pituitary tumors rarely produce metastasis, but cause considerable morbidity and mortality. Prolactinomas are the more prevalent among pituitary tumors. They are usually benign, and can be effectively treated with dopaminergic agents. Nevertheless, 15% of them may be or become resistant to classical pharmacological therapy, can be invasive, and require extirpation. In general, pituitary tumors result from monoclonal growth and intrinsic genetic defects which are related to oncogenes, suppressor genes, and genes responsible of differentiation [1]. On the other hand, growth factors of hypothalamic or pituitary origin may act on aberrant cells, contributing to their proliferation. [2]. Point mutations identified up to date can only account for a small percentage of pituitary tumors, and the mechanism of pituitary tumorigenesis is still unraveling.

Pituitary tumor transforming gene (pttg) is a recently cloned oncogene that was identified in rat pituitary tumor cells by differential mRNA display [3]. It has been proposed as an important paracrine growth factor involved in early lactotrope transformation and onset of angiogenesis in pituitary hyperplasia in estrogen-treated rats [4]. PTTG (protein encoded by pttg) has been recognized as a mammalian securin that maintains binding of sister chromatides during mitosis [5]. PTTG must be proteolysed during cell division for sister chromatid separation to occur, and failure in this process, as in PTTG overexpression, elicits inappropriate sister chromatid exchange, resulting in genetic instability as an early tumorigenic event [6].

Several lines of evidence support the role of PTTG in tumorigenesis [6,7]. Overexpressed PTTG induces cell aneuploidy, transforms NIH3T3 cells *in vitro *and *in vivo*, stimulates basic fibroblast growth factor (FGF-2) production, and stimulates proliferation and angiogenesis. Furthermore, PTTG transactivates the oncogene c-myc, which in turn may influence cell growth [6]. PTTG is expressed in high levels in different pituitary tumors [8] and other neoplasms including carcinomas of lung, breast, colon, thyroid, adrenal, liver, kidney, endometrium, uterus, ovary leukemia and lymphomas [9]. The expression of PTTG in normal tissues is restricted, with highest expression in the testis.

The role of PTTG in prolactinomas is not clear, furthermore, the relation of PTTG expression and dopamine control of pituitary function has not been established. We sought to determine the level of PTTG expression in three different experimental models of lactotrope hyperplasia with altered dopaminergic control of lactotropes: the dopaminergic D2R knockout female mouse [10], the estrogen-treated rat [11], and the senescent female rat [12]. These three models share the characteristics of a tumoral pituitary, hyperprolactinemia, lactotrope hyperplasia and reduced or absent dopaminergic action at the pituitary level [13,13-16]. We also studied human prolactinoma tissue from patients who were characterized as responsive or resistant to dopamine agonist therapy.

We believe that the elucidation of the factors involved in the regulation of lactotrope proliferation and pituitary angiogenesis might open new possibilities in the treatment of prolactinomas, especially in those cases with resistance or intolerance to dopamine agonists.

## Results

### PTTG levels in pituitaries from animal models of prolactinoma

**Figure 1:**
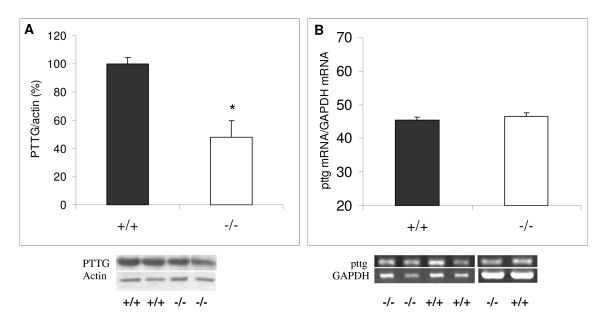
**Comparative PTTG and pttg mRNA content in pituitaries from wildtype and D2R knockout female mice**. A) Comparative pituitary PTTG content (evaluated by Western blot) in wildtype (+/+, filled bars) and D2R knockout (-/-, empty bars) female mice. For each sample, arbitrary units of band intensities for PTTG were divided by band intensities for the respective actin, and compared to those of wildtype females (considered 100%) in each series of experiments. * *P *< 0.05 vs. +/+; *N *= 12 and 15, respectively. For this and following figures, results shown are means ± SE. Below, representative western blots. B) Densitometric analysis of pituitary pttg RT-PCR products, in wildtype (filled bars) and D2R knockout (empty bars) female mice. For each sample, intensity units of the pttg band were normalized to those of the respective GAPDH band. * *P *< 0.05 vs. +/+; *N *= 5 and 5. Below representative bands are depicted.

PTTG protein and mRNA expression were determined in the lactotrope hyperplasia of the D2R female knockout mouse. When compared to female wild-type mice, pituitaries from female D2R knockout mice had decreased PTTG concentration (normalized to actin content, Figure 1A, *P *= 0.00057), while no differences in pttg mRNA expression were found (normalized to the housekeeping gene GAPDH, Figure 1B). Knockout mice had increased pituitary weight as previously described [10], and as expected, serum prolactin levels were greatly increased in knockout when compared to wildtype mice (Table 1).

**Table 1:** Pituitary weight, serum prolactin and estrogen levels in the three experimental models of prolactinomas.

	Pituitary weight (mg)	P <	serum prolactin (ng/ml)	P <	serum estrogens
Wildtype mouse	2.51 ± 0.34	0.001	29.1 ± 6.6	0.001	normal
D2R KO mouse	7.03 ± 1.39		272.8 ± 59.7		low
Young rat	11.3 ± 0.5	0.01	24.3 ± 3.2	0.01	28.9 ± 2.6 pg/ml
Senile rat	20.2 ± 6.2		98.3 ± 20.8		36.5 ± 1.8 pg/ml
Control oil- injected rat	14.7 ± 0.3	0.001	9.4 ± 1.7	0.001	normal
DES injected rat	43.6 ± 3.7		444.0 ± 41.7		high

**Figure 2:**
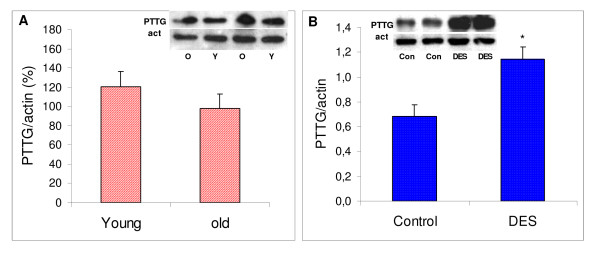
**Comparative pituitary PTTG content in young and old rats, and in control and DES-injected female rats**. Comparative pituitary PTTG content (evaluated by Western blot) in young and old rats (A: stippled bars) and control and DES-injected female rats (B: dotted bars). For each sample, arbitrary units of band intensities for PTTG were divided by band intensities for the respective actin band intensity, and compared to those of young (A) or control (B) females (considered 100%) in each series of experiments. * *P *< 0.05 vs. young or control respectively; *N *= 5 and 5 for A), and 12 and 12, for B). Inset: representative bands.

In senescent rats no difference in pituitary PTTG protein expression was found when compared to young rats (Figure 2A). Old rats had increased pituitary weight and elevated prolactin levels (Table 1). Hyperprolactinemia and hypertrophy of the gland were lower than those observed in the D2R knockout mouse.

In young female rats treated with a synthetic estrogen (DES 20 mg) pituitary weight and serum prolactin levels were increased as expected (Table 1), and PTTG protein expression was enhanced as well (*P *= 0.029, Figure 2B).

Therefore, in the three experimental models of prolactinomas pituitary size was increased and there was hyperprolactinemia, but PTTG expression followed different patterns.

### PTTG levels in macroprolactinomas from dopamine agonist responsive or resistant patients

**Figure 3:**
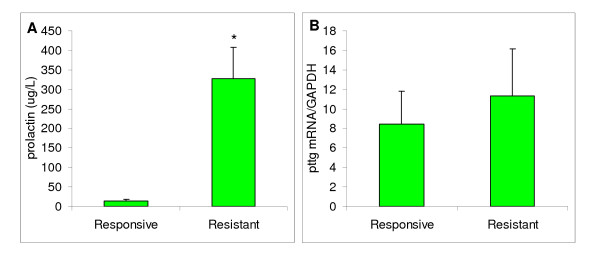
**Serum prolactin levels and PTTG mRNA in prolactinomas in dopamine agonist responsive or resistant patients**. A: Serum prolactin levels in responsive and resistant patients with prolactinomas after prolonged dopaminergic therapy (see Materials and Methods). N = 6 and 14, * *P *< 0.05. B: Comparative pituitary pttg mRNA content (evaluated by real time PCR) in patients with macroprolactinomas. N = 6 for Responsive patients (patients with normalization of prolactin levels after dopaminergic treatment), and N = 14 for patients resistant to such therapy.

Patients with prolactinomas were divided in those in whom dopaminergic therapy normalized or failed to normalize prolactin levels (responsive and resistant, respectively, Figure 3A). When pituitary pttg mRNA expression was analyzed in these macroprolactinomas, comparing resistant adenomas with those responding to dopaminergic pharmacological treatment, no differences were found (Figure 3B).

### Estrogen Receptor α levels in animal and human prolactinomas

**Figure 4:**
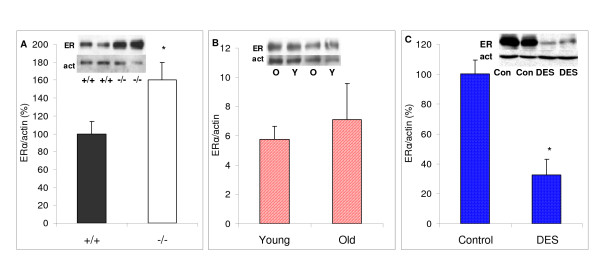
**Pituitary ERα in the experimental models of prolactinomas**. Comparative pituitary ERα content (evaluated by Western blot) in A) wildtype and D2R knockout female mice: B) Young and old female rats; and C) control and DES treated female rats * *P *< 0.05 vs. respective control. N = A) 10,9; B) 6,5; C) 7,11.

We next analyzed serum estrogen levels, and pituitary estrogen receptor α levels. In D2R knockout mice estrogen levels and action are lower than in wildtype mice and fewer estral cycles are observed; in accordance, pituitary estrogen receptors were increased (*P *= 0.047, Figure 4A). On the other hand, in senescent rats estrogen levels were slightly though not significantly higher (Young 28.9 ± 2.6 and old 36.5 ± 1.8 pg/ml, NS), and estrogen receptors were similar between groups Figure 4B).

**Figure 5:**
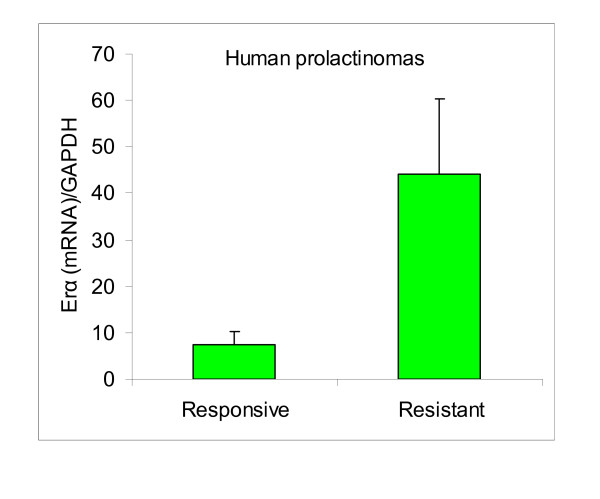
**Pituitary ERα in human responsive and resistant macroprolactinomas**. Comparative pituitary ERα mRNA evaluated by real time PCR in human responsive and resistant macroprolactinomas. N = 4,10.

The estrogen treated rats had high pharmacological levels of the synthetic estrogen, and estrogen receptors were markedly lower than in controls (*P *< 0.0001, Figure 4C). Finally, in patients with dopamine resistant prolactinomas, we found similar estrogen receptor α levels when compared to responsive tumors, even though there was a tendency to higher levels in resistant prolactinomas (Figure 5).

Evaluating the results of all groups, we observed that pituitary PTTG was increased only if estrogen action was increased, which correlated with a decrease in pituitary estrogen receptor level. We therefore conclude that PTTG does not correlate with prolactin levels in prolactinomas, and is not related to a decrease in dopaminergic control of the lactotrope, but is sensitive to estrogen action at the pituitary level.

## Discussion

Pituitary tumor-transforming gene isolated by differential display from GH-secreting pituitary tumor cell lines [3], is expressed in actively proliferating normal tissue specially the testis and lymphopoietic system, as well as in several tumor types. In particular, it is ubiquitously expressed in pituitary tumors at higher levels than those detected in normal pituitary. PTTG is required for tissue self-renewal and pttg-null mice have hypoplastic testes, spleen, and pituitary glands [17]. Furthermore, PTTG overexpression results in focal pituitary hyperplasia and adenoma formation [18].

Nevertheless the expression of pttg in prolactinomas, and in particular, in dopamine resistant prolactinomas has not been well defined. In humans, pttg overexpression in pituitary tumors has been demonstrated [19-21], but controversies on correlation between pttg levels and tumor behaviour or type exist. Higher pttg expression was detected in somatotrope tumors [19,21] and non functioning adenomas in comparison to other hormone secreting pituitary tumors [21], and pttg correlated with GH secretion in one study [19]. In another study, pttg was also highly expressed in nonfunctioning and in GH-secreting tumors but there was, in general, an ubiquitous presence of high pttg in most pituitary adenomas, and in particular, increased pttg was observed in hormone-secreting tumors that had invaded the sphenoid bone [8]. Finally, in some studies there was no correlation of pttg levels and imaged tumor volume or invasiveness [19,21].

In rats, on the other hand, pttg is clearly involved in early pituitary lactotrope tumors induced by pharmacological administration of estrogens [4].

Our results show that increased PTTG or pttg mRNA is not a common feature of lactotrope hyperplasia in experimental rodent models with decreased pituitary dopaminergic function, or in human dopamine agonist resistant compared with responsive prolactinomas. Nevertheless, PTTG protein expression is sensitive to high estrogen levels, which decreases dopaminergic control of the lactotrope.

The D2R female knockout mouse is an excellent model to study pituitary dopamine resistance. Dopamine is the principal hypothalamic inhibitory factor for lactotrope cells. Its main receptor in the pituitary is the D2R, and therefore these knockout mice develop lactotrope hyperplasia [13] followed by lactotrope tumor formation [22]. Pituitary glands of the female D2R knockout mice have markedly increased number of cells containing prolactin [23]. These lactotropes were hyperstimulated with rapid turnover of prolactin and limited storage capacity. Furthermore, we found that the expression of the angiogenic protein, vascular endothelial growth factor-A (VEGF), was increased in pituitaries from D2R knockout female mice, and increased pituitary VEGF expression was mainly dependent on the lack of dopaminergic control [24]. In the present work we show that PTTG, another angiogenic protein, is not increased in the proliferating pituitary gland of the D2R knockout mouse. This would suggest that PTTG is not regulated by dopamine. Furthermore, we found low expression of PTTG in D2R knockout compared to wildtype mice despite similar pttg transcripts. This could suggest a post-translational regulation of PTTG (e.g. an increased degradation of PTTG within the ubiquitin proteasome system in knockouts) that could be part of a protective mechanism against tumor development in the setting of lactotrope hyperplasia. On the other hand, these knockout mice have a low estrogenic environment. High prolactin levels throughout life [10] are certainly related to the relative infrequent estral cycles observed in these mice, which cease to reproduce around the fourth month of life. Therefore, low estrogenic input at the pituitary level may be insufficient to promote pttg expression.

In the female rat, we have shown that aging is characterized by a high incidence of prolactin secreting pituitary adenomas and diffuse prolactin cell hyperplasia [12,25]. These changes cannot be accounted for by different serum profiles of gonadal steroids [25]. Estrogen levels were not significantly increased in aging rats, even though we found a marked enlargement of the pituitary gland. On the other hand, aging brings about a progressive loss of both tuberoinfundibular dopaminergic system and periventricular dopaminergic system neurons [16], which may contribute significantly to lactotrope hyperplasia. There are no reports in the literature with regard to the pituitary expression of angiogenic growth factors in senescence. We found that the pituitary content of PTTG was similar in senescent and young rats, indicating that this protein is not involved in the age-related hyperplastic progression of lactotropes in aging female rats.

Chronic administration of estrogens to rats induces enlargement of the anterior pituitary and increases the synthesis and secretion of prolactin [11]. Histologically, the resulting tumors are composed of hyperplastic and hypertrophied lactotropes [26]. Damage to hypothalamic dopaminergic neurones in response to estrogen [14] as well as a direct action of estrogen at the pituitary level decreasing dopamine sensitivity [27,28] have been proposed as causative factors. Besides, the effect of estrogen can be accounted for by alterations in different growth factors: VEGF, and FGF-2 [4,29,30], even though increases in such growth factors do not necessarily imply their strict requirement for tumor development [31]. In accordance with the literature, we describe a marked increase in pituitary PTTG expression in the estrogen-treated rats.

PTTG was initially proposed as an angiogenic and/or oncogenic factor in human pituitary tumors [32]. In fact, abundant and concordant PTTG and bFGF expression in different human pituitary tumors was described [4]. Furthermore, anti-estrogens reduced PTTG expression in human pituitary tumors *in vitro *(three gonadotroph, five nonfunctioning) and suppressed experimental tumor growth *in vivo *[29]. But, we describe that PTTG expression is not increased in human dopamine resistant when compared to dopamine responsive prolactinomas. This is consistent with experimental animal results presented herein which demonstrate that reduced dopaminergic action at the pituitary is associated with pituitary enlargement, hyperprolactinemia but not necessarily with increased PTTG expression.

Therefore, taken together, our results suggest that PTTG increment in estrogen-induced prolactinomas is not related to the deficient dopaminergic control of the pituitary found in this model, but to another estrogenic pathway.

We measured pituitary estrogen receptors in order to establish a link with circulating estrogen and pituitary PTTG expression. Cellular estrogen receptor levels are dynamic and are particularly sensitive to changes in circulating levels of 17β-estradiol. The level of steroid receptors in cells changes with varying physiological states. In most cases, the primary endocrine regulator is the ligand itself. In an autoregulatory feedback loop, estrogen induces a decline in both ERα protein and mRNA in lactotrope cells. It has been demonstrated through a number of studies that the decline in ERα upon exposure to 17β-estradiol results from a combination of mechanisms that control both receptor synthesis and degradation through transcriptional, posttranscriptional, and posttranslational mechanisms [33,34]. The most rapid of these regulatory mechanisms is the direct loss of ERα protein brought about by the induction of proteasome-mediated proteolysis [35]. Our results show that the only experimental model with low pituitary estrogen receptors was the estrogen-treated rat, indicating a down regulating effect of the pharmacological administration of the synthetic estrogen. On the other hand, in the pituitary of the D2R knockout female mouse estrogen receptors were high when compared to their wildtype counterparts, indicating reduced serum levels of estradiol which correlate with the reduced fertility of these mice, and with results of another D2R knockout mouse [36]. In aging rats neither pituitary estrogen receptor levels nor serum estrogen levels were significantly different from young rats. And finally, in the pituitaries from human prolactinomas estrogen receptors were not significantly different between responsive and resistant tumors.

So even though PTTG expression is increased in rapidly proliferating cells in some experimental models supporting that it may play a role in the control of cell proliferation, we postulate that it does not participate in the prolactinoma development caused by deficient dopamine control of the lactotrope. Emerging data clearly indicate that different molecular mechanisms are involved in the pathogenesis of the various pituitary tumor subtypes, and PTTG may be increased in somatotropinomas and other pituitary tumors, but not in dopamine resistant in comparison to dopamine sensitive prolactinomas, and it is not regulated by dopamine in different experimental prolactinomas. On the other hand, high estrogen levels could impact on PTTG expression. Finding reliable prognostic indicators for patients with pituitary adenomas will make tumor-specific therapy possible, and elucidation of the particular oncogenes or growth factors involved in each pituitary tumor subtype will be fundamental to determining the poorly understood phenomenon of pituitary tumorigenesis.

## Methods

### Animals

#### A) D2 dopamine receptor female knockout mice (KO)

(official strain designation B6; 129S2-*Drd2*^*tm1low *^by the Induced Mutant Resource at The Jackson Laboratory, Bar Harbor, ME), generated by targeted mutagenesis of the D2R gene in embryonic stem cells [13,22] were used. The original F_2 _hybrid strain (129S2/Sv × C57BL/6J) containing the mutated D2R allele was backcrossed for eight generations to wild-type C57BL/6J mice (WT). Mutant and wild-type mice were generally the product of heterozygote crossings, and in all cases sibling controls were used. Mice were housed in groups of 4 or 5 with mixed genotypes in an air-conditioned room with lights on at 0700 and off at 1900 h. They had free access to laboratory chow and tap water. Wild-type, heterozygous and knockout mice were identified by PCR of genomic DNA, as previously described [10]. Animals were used at 6–8 months. All experimental procedures were reviewed and approved by the institutional animal care and use committee of the Instituto de Biología y Medicina Experimental, Buenos Aires (Division of Animal Welfare, Office for Protection of Research Risks, National Institutes of Health, A#5072-01).

#### B) Senescent rats

Young (5 mo) and senescent (28–31 mo) female Sprague-Dawley rats, raised in the gerontological rat colony of INIBIOLP, Universidad de La Plata, Buenos Aires, were used. Animals were housed in a temperature-controlled room (22 ± 2°C) on a 14:10 h light/dark cycle. Food and water were available *ad libitum*. In our colony, the average 50% survival time for females, studied in groups of 50–60 animals, is 33 months (range 32–34 mo).

#### C) Rats with DES treatment

Female 60-day-old Sprague-Dawley rats were divided into two treatment groups (each of seven animals). Pituitary tumors were induced by sc implantation of a 20 mg pellet of diethylstilbestrol (DES group) (Sigma Mo) for seven weeks, in one group, and the other group was used as control.

### Patients with dopamine responsive and resistant prolactinomas

Following ethical approval and patient consent, tissue of 20 macroprolactinomas from patients submitted to surgery were studied (14 women and 6 men, mean age at diagnosis 23.4 ± 1.7 and 39.5 ± 4.4, respectively). The removed tissues were quickly frozen in liquid nitrogen and stored at -80°C for subsequent molecular studies. Patients were followed at the Hospital das Clinicas, University of Sao Paulo Medical School, Sao Paulo, Brazil. Before starting dopaminergic treatment, average serum prolactin level was 2302 μg/L, ranging from 70.0–16000 μg/L. Patients were divided as responsive (N = 6) and resistant (N = 14) to dopamine agonist therapy, taking in consideration prolactin normalization after pharmacological treatment. The 3 responsive patients in whom follow up data was available were treated for 55 months (range 3–83 mo) with a mean bromocriptine dose of 10.83 mg/day (range, 5–17.5 mg/day). In these patients the mean lower prolactin levels during treatment was 11.03 ug/L. Resistant patients were treated for 20,5 months (range 3–63 months), and the mean lowest prolactin levels during treatment was 306.95 μg/L. Surgery indication, besides prolactinoma dopamine agonist resistance were tumor expansion, visual field defects and headache, or intolerance to medical treatment

#### Drugs

Unless otherwise specified, all chemicals were purchased from Sigma (St. Louis, MO).

#### RIAs

Mouse and rat serum prolactin were measured by RIA using kits provided by the National Institute of Diabetes and Digestive and Kidney Diseases [NIDDK; Dr. A.F. Parlow, National Hormone and Pituitary Program (NHPP), Torrance, CA]. Results are expressed in terms of mouse prolactin RP3 and rat prolactin RP3. Intra- and interassay coefficients of variation were 7.2% and 12.8%, and 6.5% and 11.5% respectively.

17β-estradiol was measured by RIA using commercial solid phase kits (Coat-A-Count, DPC, Los Angeles, CA)

Human serum PRL was determined between 0800 and 1100 h, by a immunofluorimetric assay (Wallac AutoDELFIA. PerkinElmer Life Sciences. Boston, MA). The normal references values ranged from 2 to 10 μg/liter for men and 2 to 15 μg/liter for women. Intra- and interassay coefficients of variation were 1.05 and 2.60%, respectively

#### Western blot

Anterior pituitaries from mice or rats were homogenized in 80 μl ice-cold buffer containing 60 mM Tris-HCl, 1 mM EDTA (pH 6.8) and a mix of protease inhibitors (phenyl-methyl-sulphonile, TPCK, TAME, ZPCK and TLCK) in a handheld microtissue homogenizer. The homogenate was then centrifuged at 800 × g for 5 min at 4°C. An aliquot of supernatant was taken to quantify proteins by the Lowry method. Thirty micrograms of proteins in 10 μl of homogenization buffer were mixed with 10 μl 2× sample buffer (60 mM Tris-HCl, 4 % sodium dodecyl sulfate (SDS), 20 % glycerol, 0.02% bromophenol blue and 50 mM dithiotreitol (pH 6.8). Samples were sonicated during 20 sec, heated 5 min at 95°C and subjected to 12% SDS-polyacrylamide gel electrophoresis. The gel was then blotted onto a nitrocellulose membrane (Bio-Rad) and probed with the corresponding primary antibody followed by a secondary antibody conjugated with horseradish peroxidase. Polyclonal rabbit pttg antibody (1:1,000, sc-5846, Santa Cruz Biotechnology or antibody provided by S.S. Kakar) was used. Estrogen receptor-α (ERα MC-20, sc-542 was purchased from Santa Cruz Biotechnologies). Mouse monoclonal actin antibody (Ab-1) was purchased from Labvision Co. (Freemont, CA). Immunoreactive proteins were detected by enhanced chemoluminiscence (Amersham, Aylesbury, UK). For repeated immunoblotting, membranes were incubated in stripping buffer (62.5 mM Tris, 2 % SDS and 100 mM mercaptoethanol, pH 6.7) for 40 min at 50°C and reprobed. Band intensities were quantified using the ImageQuant software.

#### Preparation of mouse pituitary RNA

Total RNA was isolated from anterior pituitaries using TRIzol Reagent (Gibco). Each gland was homogenized in 100 μl TRIzol, sonicated for 10 sec, and incubated at room temperature for 5 min. Chloroform (20 μl) was added, samples were shaken vigorously, and after 5 min of incubation at room temperature, they were centrifuged at 12,000 × g for 15 min at 4°C. Isopropanol (50 μl) was added to the supernatant to precipitate the RNA. After 10-min incubation at room temperature, samples were centrifuged at 12,000 × g for 10 min at 4°C, supernatants discarded, and their pellets washed with 100 μl of 70% ethanol. The resulting precipitates were resuspended in 5 ul diethylpyrocarbonate-treated water. RNA was quantified by UV spectrophotometry and its integrity checked by gel electrophoresis.

#### Semiquantitative RT-PCR for mouse samples

Total RNA (150 ng) was reverse transcribed in a reaction mixture containing 50 mM Tris-HCl (pH 8.3), 75 mM KCl, 3 mM MgCl_2_, 10 mM dithiotreitol, 1 mM deoxynucleotide triphosphates (dNTPs), 8 U RNAse inhibitor (Promega, Madison, WI), 1 μg of random hexamers (Biodynamics SRL, Buenos Aires, Argentina) and 200 U Moloney murine leukemia virus transcriptase (Invitrogen Life Technologies, Buenos Aires, Argentina) in a final volume of 20 μl. After incubation at 37°C for 60 min, samples were heated for 10 min at 70°C to inactivate the transcriptase. The product was amplified with mouse pttg and GAPDH sense and antisense primers in a reaction mixture (30 μl) containing 2 mM Tris-HCl (pH 8.0), 1.5 mM KCl, MgCl_2_, 0.2 mM dNTPs, 0.33 μM of each primer, and 2.5 U *Taq *DNA polymerase (Invitrogen Life Technologies), using an Eppendorf thermal cycler.

In Table 2 primer sequences are detailed. Common steps were a hot start step of 3 min at 95°C, followed by n cycles of denaturation at 94°C for 60 sec, annealing at an adequate temperature (see Table 2) for 60 sec, and extension at 72°C for 50 sec, with a final elongation step of 5 min at 72°C.

**Table 2:** Gene-specific primers for mouse PTTG and GAPDH, and human PTTG, Estrogen receptor α and GAPDH used in this study

Gene	Sequence (Forward)	Nucleotide location	Sequence (reverse)	Nucleotide location	Product size bp	Cycles	Temp annealing
Mouse PTTG	CAGCCGTGCCTAAAGCCAGC	463–482	GATAGAAAGGGTGTCTTCAGAG	799–820	358	35	58°C
Mouse GAPDH	CTCACGGCAAATTCAACGG	195–213	CTTTCCAGAGGGGCCATCCA	603–622	428	26	55°C
Human PTTG1	CGATGCCCCACCAGCCTTACC	195–215	CAAGCTCTCTCTCCTCGTCAAGG	489–511	317	40	57°C
Human ER-α	TCCAGCACCCTGAAGTCTCT	1749–1768	TCTCCAGCAGCAGGTCATAG	1970–1989	241	40	63°C
Human GAPDH	GCCAAAAGGGTCATCTC	271–290	GCAGGGATGATGTTCTGGAG	532–551	281	32	57°C

Preliminary experiments using various RNA concentrations and cycle numbers confirmed that these PCR reactions were performed within the linear phase of the PCR amplification reaction and the amplified product was analyzed by 1.8 % agarose gel electrophoresis. Bands were detected by ethidium bromide staining. Densitometric analysis was conducted using the Scion Image software and intensity values of pttg PCR products were normalized to the corresponding GAPDH products.

### RNA Isolation and Reverse Transcriptase Reaction from human samples

Tumor tissue stored in liquid nitrogen was fragmented in a tissue pulverizer (Mikro-Dismembranator, B. Braun Biotech International, Melsungen, Germany). Total RNA was extracted from approximately 100 mg tissue after homogenization, using the Trizol reagent (Invitrogen, Carlsbad, USA) according to the manufacturer's recommendations. The RNA concentration (1.7–1.8) was measured by UV spectrophotometry at 260 nm. Only samples with OD260/280 ratio >1.7 were further processed. RNA integrity was assessed by 1% agarose gel electrophoresis in the presence of ethidium bromide.

One μg total RNA was then retrotranscribed into a reaction mixture (40 μl) containing 100 U Moloney murine leukemia virus reverse transcriptase (Amersham Pharmacia Biotech,), 1× reaction buffer (Amersham), 0.2 mM deoxynucleotide triphosphates (Promega,), 50 pmol oligo(dT) primers (Invitrogen, Life technologies), 20 U ribonuclease inhibitor (Promega). The reaction was carried out at 37°C for 60 min then at 95°C for 5 min.

#### Real-Time PCR

Gene-specific primers for tested genes are presented in Table 2. The relative quantification was given by the Ct values, determined in duplicate reactions for test and reference samples for the target and for the internal control gene (GAPDH). Duplicate Ct values were averaged and the GAPDH subtracted to obtain DCt [DCt = Ct (target gene) - Ct (GAPDH gene)]. DCt values were calculated for each gene and reference sample. Relative expression level was determined as 2-DDCt, where DDCt = DCt (target sample) - DCt (reference sample). For the reference sample, DDCt equals 0 and 20 equals 1, so the fold change in the reference sample equals 1 by definition. For the unknown samples, evaluation of 2-DDCt indicates the fold change in gene expression relative to the reference sample. Values were expressed as n-fold differences in target gene expression. The reactions were prepared according to standard protocols for one-step QuantiTect SYBR Green RT-PCR (Qiagen): SYBR Green amplification mixtures (20 μl) contained 10 μL of 2× QuantiTect SYBR Green RT-PCR Master Mix, 1.0 μM of each forward and reverse primer, 0.2 μL of QuantiTect RT and 80 ng template DNA. The cycling conditions were as follows: 30 min at 50°C, 95°C for 15 min, 40–50 cycles at 95°C for 20 s, 55–63°C for 30 s and 72°C for 30 s before a final primer sequence extension incubation at 72°C or 5 min (number of cycles and annealing temperatures differed according to the gene as shown in Table 2). Primers were selected using the computer program Prime3, and designed to span at least one intron region to avoid genomic DNA amplification.

### Statistical analyses

Results are expressed as means + SEM. T-test was used. *P *< 0.05 was considered significant.

## Conclusion

We conclude that PTTG does not correlate with prolactin levels or tumor size in animal models prolactinoma, and its pituitary content is not related to a decrease in dopaminergic control of the lactotrope, but to estrogen action at the pituitary level. Therefore it is increased only in prolactinomas generated by estrogen treatment, and not in prolactinomas arising from deficient dopamine control, or in dopamine resistant compared with dopamine responsive human prolactinomas. These results are important in the search for reliable prognostic indicators for patients with pituitary adenomas which will make tumor-specific therapy possible, and help to elucidate the poorly understood phenomenon of pituitary tumorigenesis.

## Competing interests

The author(s) declare that they have no competing interests.

## Authors' contributions

CC: carried out the molecular studies in knockout mice, and drafted the manuscript; GDT: participated in the design of the study and performed the statistical analysis of animal models; RGG: carried out molecular studies in the old rats; SSK: participated in the determination of PTTG by Western blot carried out using the antibody developed by him, and revised critically the manuscript for important intellectual content; MPM: carried out studies with estrogen treated rats; VQP: participated in the acquisition of data, and in performing the molecular studies in dopamine resistant and responsive patients; DGN: was involved in the analysis and interpretation of data in dopamine resistant and responsive patients; MDB was involved in the revising of the manuscript critically for important intellectual content; DBV conceived the collaborative study, participated in its design and coordination and helped to draft the manuscript. All authors read and approved the final manuscript.
